# Added Value of SPECT/CT in Radio-Guided Occult Localization (ROLL) of Non-Palpable Pulmonary Nodules Treated with Uniportal Video-Assisted Thoracoscopy

**DOI:** 10.3390/jcm14155337

**Published:** 2025-07-29

**Authors:** Demetrio Aricò, Lucia Motta, Giulia Giacoppo, Michelangelo Bambaci, Paolo Macrì, Stefania Maria, Francesco Barbagallo, Nicola Ricottone, Lorenza Marino, Gianmarco Motta, Giorgia Leone, Carlo Carnaghi, Vittorio Gebbia, Domenica Caponnetto, Laura Evangelista

**Affiliations:** 1 Nuclear Medicine Unit, Humanitas Istituto Clinico Catanese, 95045 Catania, Italy; 2 Medical Oncology Unit, Humanitas Istituto Clinico Catanese, 95045 Catania, Italy; 3 Department of Clinical and Experimental Medicine, University of Catania, 95124 Catania, Italy; 4 Thoracic Surgery Unit, Humanitas Istituto Clinico Catanese, 95045 Catania, Italy; 5 Radiotherapy Unit, Humanitas Istituto Clinico Catanese, 95045 Catania, Italy; 6 Pathology Section, Humanitas Istituto Clinico Catanese, 95045 Catania, Italy; 7 Faculty of Medicine and Surgery, “Kore” University of Enna, 94100 Enna, Italy; 8 Data Manager Unit, Humanitas Istituto Clinico Catanese, 95045 Catania, Italy; 9 Department of Biomedical Science, Humanitas University, 20089 Milan, Italy

**Keywords:** SPECT/CT, ROLL, VATS, minimally invasive surgery, solitary pulmonary nodules

## Abstract

**Background/Objectives:** The extensive use of computed tomography (CT) has led to a significant increase in the detection of small and non-palpable pulmonary nodules, necessitating the use of invasive methods for definitive diagnosis. Video-assisted thoracoscopic surgery (VATS) has become the preferred procedure for nodule resections; however, intraoperative localization remains challenging, especially for deep or subsolid lesions. This study explores whether SPECT/CT improves the technical and clinical outcomes of radio-guided occult lesion localization (ROLL) before uniportal video-assisted thoracoscopic surgery (u-VATS). **Methods:** This is a retrospective study involving consecutive patients referred for the resection of pulmonary nodules who underwent CT-guided ROLL followed by u-VATS between September 2017 and December 2024. From January 2023, SPECT/CT was systematically added after planar imaging. The cohort was divided into a planar group and a planar + SPECT/CT group. The inclusion criteria involved nodules sized ≤ 2 cm, with ground glass or solid characteristics, located at a depth of <6 cm from the pleural surface. ^99m^Tc-MAA injected activity, timing, the classification of planar and SPECT/CT image findings (focal uptake, multisite with focal uptake, multisite without focal uptake), spillage, and post-procedure complications were evaluated. Statistical analysis was performed, with continuous data expressed as the median and categorical data as the number. Comparisons were made using chi-square tests for categorical variables and the Mann–Whitney U test for procedural duration. Cohen’s kappa coefficient was calculated to assess agreement between imaging modalities. **Results:** In total, 125 patients were selected for CT-guided radiotracer injection followed by uniportal-VATS. The planar group and planar + SPECT/CT group comprised 60 and 65 patients, respectively. Focal uptake was detected in 68 (54%), multisite with focal uptake in 46 (36.8%), and multisite without focal uptake in 11 patients (8.8%). In comparative analyses between planar and SPECT/CT imaging in 65 patients, 91% exhibited focal uptake, revealing significant differences in classification for 40% of the patients. SPECT/CT corrected the classification of 23 patients initially categorized as multisite with focal uptake to focal uptake, improving localization accuracy. The mean procedure duration was 39 min with SPECT/CT. Pneumothorax was more frequently detected with SPECT/CT (43% vs. 1.6%). The intraoperative localization success rate was 96%. **Conclusions:** SPECT/CT imaging in the ROLL procedure for detecting pulmonary nodules before u-VATs demonstrates a significant advantage in reclassifying radiotracer positioning compared to planar imaging. Considering its limited impact on surgical success rates and additional procedural time, SPECT/CT should be reserved for technically challenging cases. Larger sample sizes, multicentric and prospective randomized studies, and formal cost–utility analyses are warranted.

## 1. Introduction

In recent decades, advances in computed tomography (CT) and lung cancer screening programs have significantly increased the detection of small pulmonary nodules previously undetectable with conventional imaging [1,2,3]. While CT and positron emission tomography (PET) offer high diagnostic accuracy, they often require histological confirmation for definitive lesion characterization [4]. This challenge has led to an increase in biopsy procedures to obtain tissue samples. However, many pulmonary nodules are smaller than 1 cm, subsolid in nature, and located deep within the lung parenchyma, making biopsies frequently inconclusive and necessitating a surgical approach [5]. Advancements in surgical techniques have facilitated minimally invasive approaches, such as video-assisted thoracoscopic surgery (VATS). VATS is considered the preferred method for performing surgical wedge resections, segmentectomies, and lobectomies [6]. Additionally, single-port or uniportal VATS (u-VATS) has demonstrated high efficacy and lower perioperative morbidity compared to multiport VATS (m-VATS), making it an increasingly favored approach [7]. A major limitation of VATS—regardless of uni- or multiportal access—is the difficulty of accurately locating small or impalpable pulmonary nodules, particularly those positioned deep beneath the pleural surface [8,9]. Intraoperative localization without guidance is often challenging and may necessitate conversion to open thoracotomy, thereby negating the advantages of thoracoscopy, such as reduced operative time and lower perioperative morbidity [7]. Therefore, precise localization of the nodule is crucial for a successful resection [10].

Several preoperative localization techniques have been developed to enhance the intraoperative detection of pulmonary nodules during VATS. These include the percutaneous injection of liquid markers such as methylene blue or lipiodol, the placement of metallic markers such as microcoils or hook wires, and advanced imaging techniques including image-guided navigation and intraoperative ultrasound [11,12,13]. While each technique has its advantages and limitations, the lack of randomized clinical trials has resulted in an absence of standardized guidelines, with current evidence based solely on case series [14]. Since September 2017, our institute has adopted the CT-guided percutaneous injection technique for radio-guided occult lesion localization (ROLL) with ^99m^Tc-Macroaggregated Albumin (MAA), initially described by Chella et al. and later refined by Grogan et al. in 2008 and Bellomi et al. in 2010 [15,16,17]. A recent review has highlighted ROLL as a viable alternative to existing localization methods, such as fluoroscopy, intraoperative ultrasound, and hook wire placement, due to its low cost, minimal morbidity, ability to maintain localization accuracy over time, low radiation exposure for both patients and operators, and short procedural duration [18]. Our previous study confirmed that ROLL is both effective and safe in patients undergoing u-VATS, achieving a high detection rate for solitary pulmonary nodules. Additionally, the preliminary findings suggested that SPECT/CT imaging could enhance the accuracy of this procedure by confirming the presence of ^99m^Tc-MAA within the target nodule and identifying potential spillage into the pleura, bronchial tree, or gastrointestinal tract [19]. However, the available literature on the role that SPECT/CT plays in this context remains limited [20], thus warranting further investigation.

Therefore, the objective of this study was to evaluate whether SPECT/CT improves the technical and clinical outcomes of ROLL before uVATs.

## 2. Materials and Methods

### 2.1. Study Design

This is a retrospective, single-center study that included patients with non-palpable small pulmonary nodules who underwent the ROLL procedure followed by u-VATS between September 2017 and December 2024. All patients provided their written informed consent after receiving detailed information about the study procedures. Ethical review and approval were waived for this study due to retrospective study.

### 2.2. Patient Selection

All patients were referred to the thoracic surgery department for evaluation and management of a small pulmonary nodule. The inclusion criteria were as follows: nodule size ≤ 2 cm, ground glass or solid radiological features, and depth < 6 cm from the pleural surface. Exclusion criteria: age < 18, known allergy to lidocaine or albumin derivatives, presence of multiple pulmonary nodules, severe COPD, and poor compliance. These criteria were applied to ensure procedural safety and data consistency, in line with the current recommendations in the literature [21,22]. Patient selection was conducted through a multidisciplinary board discussion involving thoracic surgeons, radiologists, medical oncologists, nuclear medicine physicians, pathologists, and radiation oncologists. The board assessed the diagnostic strategy and feasibility of percutaneous radiopharmaceutical injection under CT guidance, comparing uniportal VATS (u-VATS) with open thoracotomy. The surgical approach—wedge resection for superficial nodules or anatomic segmentectomy for deeper lesions—was determined based on the distance of the lesion from the pleura (cut-off 2 cm) and hilar structures.

### 2.3. Techniques

**Figure 1:**
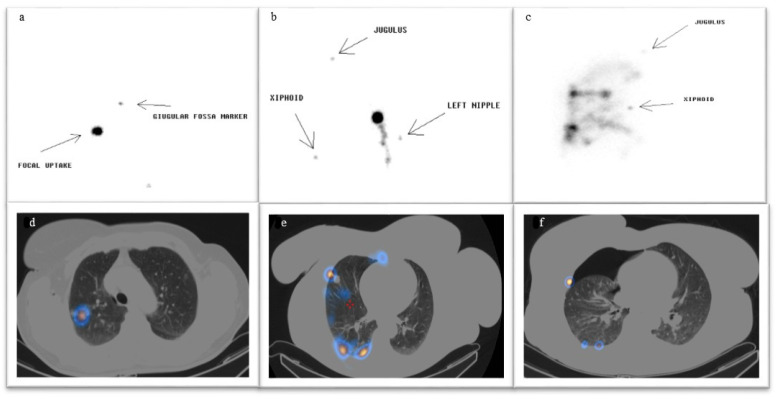
Planar imaging: (**a**) **f**ocal uptake; (**b**) multisite with focal uptake. (**c**) Multisite without focal uptake. SPECT/CT imaging: (**d**) focal uptake; (**e**) multisite with focal uptake; (**f**) multisite without focal uptake and pnx. Definitions: focal uptake: only target was visible; multisite with focal uptake. Some areas of uptake were visible in both planar and SPECT/CT images, but the target was individualizable; multisite without focal uptake. Some areas of uptake were visible in both planar and SPECT/CT images, and the target was not visible.

ROLL: On the day of surgery, all patients underwent ROLL within the Nuclear Medicine Department. A baseline CT scan was performed using the CT component of a hybrid PET/CT scanner (Discovery 690, GE Healthcare, Milwaukee, WI, USA) to determine the optimal needle entry point. The factors considered for trajectory selection included skin-to-nodule distance, the presence of intervening bony or muscular structures, and the position of pulmonary fissures to avoid interlobar needle passage. Under local anesthesia, a 21-gauge Chiba needle was inserted under CT guidance and positioned either within or immediately adjacent to the nodule. A solution containing 0.1–0.2 mL of ^99m^Tc-Macroaggregated Albumin (MAASOL; GE Healthcare, Chalfont St. Giles, UK) and 0.1–0.2 mL of non-ionic contrast medium was injected. A post-injection CT scan confirmed the intranodular or peri-nodular distribution of the radiotracer and ruled out complications such as pneumothorax or significant hemorrhage. Planar imaging (anterior and lateral projections, 70 kcounts, 256 × 256 pixels, zoom 1.33) was acquired using a hybrid gamma camera (NM-CT 800; GE Healthcare, Milwaukee, WI, USA or Symbia; Siemens Healthcare, Colorado Springs, CO, USA) to verify radiotracer localization and detect potential extravasation (Figure 1). Since January 2023, planar imaging has been supplemented with SPECT/CT imaging (matrix 128 × 128, FOV 40 cm, step-and-shoot, view angle 6°, 3″/step, body-contouring; CT: helical scan, 120 kV, 90 mA, thickness 3.75 mm, pitch 1.675 mm/rotation; 512 matrix, dFOV 50 cm) to enhance the localization accuracy of ^99m^Tc-MAA within the target nodule. In the SPECT/CT protocol, an additional CT scan was performed approximately 10 min after the CT-guided radiotracer injection, allowing for the better detection of potential pneumothorax and spillage.

### 2.4. Surgical Procedure

Intraoperatively, a gamma probe (Neo 2000; Neoprobe, Cincinnati, OH, USA) was used to localize the nodule on the lung surface based on both numerical (kcounts) and acoustic signal intensity. During lung resection, the gamma probe was used repeatedly to ensure the maximal radiotracer signal within the resected specimen. The wedge resection was performed, and the specimen was retrieved in an endo-bag. Post-resection, the gamma probe was used to confirm the absence of significant residual radioactivity within the lung parenchyma and to identify the region of highest activity in the excised specimen. An intraoperative frozen-section histopathological examination was performed, and the extent of resection was adjusted accordingly.

### 2.5. Data Collection

The patient’s demographics and nodule characteristics on pre-procedural CT scans, including size (in mm), volume (in cm^3^), density (ground glass opacity, partial solid ground glass opacity or solid), lobar localization, and depth from the pleural surface (in mm), were collected. Moreover, data from the nuclear medicine procedure, including ^99m^Tc-MAA injected activity, timing, and the classification of planar and SPECT/CT image findings, were taken. Tracer distribution was classified prospectively as follows: focal uptake as a single, well-defined hotspot corresponding anatomically to the intended nodule site; multisite with focal uptake, as multiple areas of radiotracer activity, but with one clearly dominant focus at the target nodule; and multisite without focal uptake, as a scattered or diffuse tracer distribution without a clear dominant hotspot matching the nodule location.

These categories were applied by two blinded nuclear medicine physicians using fused CT and scintigraphic images. Spillage (pleural space, bronchial or gastrointestinal tract) and post-procedure complications (pneumothorax or hemorrhage) were also considered. Figure 1 shows the planar and SPECT/CT classification finding. The surgery procedure included intraoperative gamma probe localization and timing.

### 2.6. Outcome Measure

Postoperative thoracic CT and histopathology were used to confirm the effectiveness of the u-VATS resection. Successful resection was defined as the absence of residual tumor nodules on postoperative imaging and negative surgical margins with no residual malignancy in the lung parenchyma.

### 2.7. Statistical Analysis

A descriptive analysis was made for all patients. Continuous data were expressed as the median (range) and categorical data as the number (percentages). Based on prior experience with planar scintigraphy/CT in ROLL procedures, a complication rate of 6% was observed, with a hypothesized increase to 20% using SPECT/CT, necessitating a sample size of 50 patients for adequate statistical power (α = 0.05, β = 0.20). A comparison between categorical variables, such as planar findings vs. SPECT findings, was made by using a contingent table; chi-square analysis was adopted. The distribution of continuous variables was assessed using the Shapiro–Wilk test. Since the data deviated from a normal distribution, the Mann–Whitney U test was applied for comparing independent groups. Categorical variables were compared using the chi-square test or Fisher’s exact test when appropriate. A *p*-value < 0.05 was considered statistically significant. Agreement between the two imaging modalities was assessed using Cohen’s kappa coefficient (κ), which quantifies inter-rater reliability by accounting for agreement beyond chance. The kappa statistic was interpreted as follows: <0.20, poor agreement; 0.21–0.40, fair agreement; 0.41–0.60, moderate agreement; 0.61–0.80, substantial agreement; and 0.81–1.00, almost perfect agreement. Heatmaps were generated to visualize the distribution of patients across the different classification categories. A *p*-value < 0.05 was considered statistically significant. Statistical analyses were performed using GraphPad Prism (Version 9, GraphPad Software, San Diego, CA, USA).

## 3. Results

**Figure 2:**
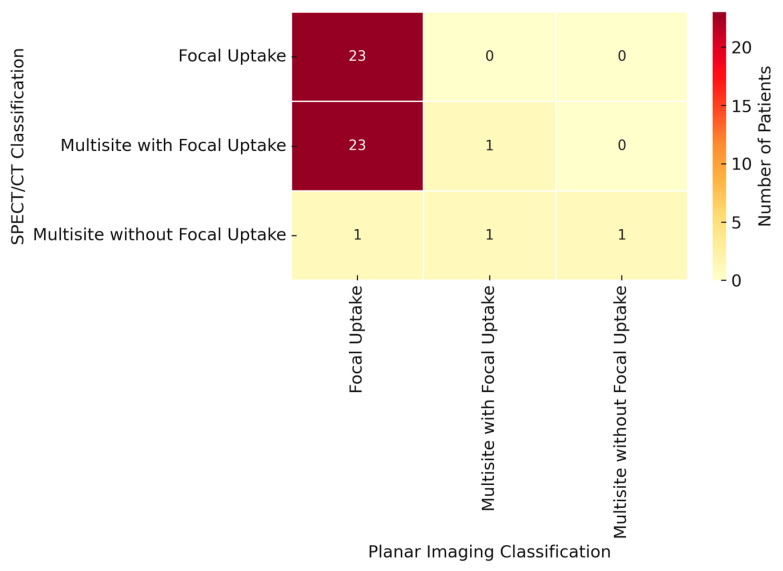
Heatmap of planar versus SPECT classification. This heatmap represents the distribution of patients according to planar and SPECT classifications. The number of patients in each category is shown in the corresponding cell. The intensity of color reflects the frequency, with darker shades indicating a higher number of patients.

Following a multidisciplinary decision, 125 patients were selected to undergo CT-guided radiotracer injection for small lung nodules, followed by u-VATS. There were no missing data among 125 patients. Table 1A presents the main clinical and demographic characteristics of the patients. In order to address potential baseline imbalances between the planar and planar + SPECT/CT groups, a comparative analysis of the clinical and radiological characteristics was performed (Table 1B). No statistically significant differences were found in terms of age (66 vs. 68 years; *p* = 0.28), sex (M/F: 28/32 vs. 27/38; *p* = 0.70), or nodule depth (16.1 mm vs. 13.7 mm; *p* = 0.09). However, the SPECT/CT group included nodules with a significantly larger median diameter (12 mm vs. 9 mm; *p* < 0.001) and volume (90.5 mm^3^ vs. 38.2 mm^3^; *p* < 0.001), and a higher frequency of ground glass and part-solid components (*p* = 0.002). These differences were not due to selective assignment, as SPECT/CT was systematically introduced for all patients starting from January 2023, independent of lesion morphology. Planar imaging was performed in all patients, revealing focal uptake in 68 out of 125 (54%), multisite with focal uptake in 46 out of 125 (36.8%), and multisite without focal uptake in 11 out of 125 patients (8.8%). The correct positioning of ^99m^Tc-MAA on planar images was confirmed in 114 patients (91.2%). In 65 out of 125 patients, both planar and SPECT/CT images were acquired. In these latter subsets of patients, focal uptake was shown in 59 patients (91%), multisite with focal uptake in 4 patients (6%), and multisite without focal uptake in 1 patient (1.5%). No data were obtained for one patient (1.5%). A comparative analysis between the planar imaging and SPECT imaging reclassified 26 procedures (40%) with statistically significant differences (*p <* 0.0001; Figure 2). In particular, 23 patients initially classified as having multisite with focal uptake on planar imaging were reclassified as having focal uptake on SPECT imaging. Additionally, two patients initially categorized as having multisite without focal uptake on planar imaging were reclassified on SPECT imaging as having focal uptake and multisite with focal uptake, respectively. Furthermore, in 2 out of 65 patients, the placement of ^99m^TcMAA was considered to be incorrect. In the first patient, both planar and SPECT/CT images showed ^99m^Tc-MAA pleural spillage, classified as multisite without focal uptake. In the second patient, planar imaging initially showed focal uptake, suggesting a successful procedure. However, the SPECT/CT images revealed that this focal uptake corresponded to the chest wall rather than the pulmonary nodule, leading to the procedure being classified as missing based on the SPECT/CT findings. To evaluate the agreement between the two imaging methods, we calculated Cohen’s kappa coefficient, which yielded a value of 0.14, indicating poor agreement beyond chance. This result suggests that SPECT/CT imaging often reclassifies patients compared to the planar approach, particularly in cases initially categorized as “planar multisite with focal” (Figure 2). The differences between planar imaging and SPECT/CT are summarized in Table 2. We also compared the median duration of the nuclear medicine procedure, and we observed that using only planar imaging, the duration was 30 min (range: 16–131 min), whereas with SPECT/CT imaging, it was 39 min (range: 18–82 min). This difference was statistically significant (*p <* 0.005). Similarly, the mean duration of the surgical procedure was 1 h and 47 min (range: 25 min to 4 h and 35 min) with only planar imaging compared to 2 h and 15 min (range: 35 min to 5 h and 10 min) with SPECT/CT acquisition (*p <* 0.005). In addition, we also compared the occurrence of radiotracer spillage during the procedure, assessing the differences between planar and SPECT/CT imaging. With the planar technique, pleural and tracheo-bronchial spillage were observed in 48.3% and 1.6% of cases, respectively. In contrast, with SPECT/CT acquisition, pleural spillage was detected in 17%, subcutaneous spillage in 1.5%, tracheo-bronchial spillage in 24.6%, and gastrointestinal spillage in 1.5% of cases. This difference in spillage between the two techniques was statistically significant (*p <* 0.0001). Pneumothorax was the most frequent complication, occurring in 23.2% of cases. It was more often detected on SPECT/CT images (43%) compared to planar images (1.6%), with a statistically significant difference (*p* < 0.0001). Another possible complication was hematoma, which was detected in only one case using SPECT/CT imaging. The overall success rate of ROLL was 96%, as confirmed by postoperative thoracic CT and histopathology, which demonstrated the absence of residual tumor nodules on imaging and negative surgical margins with no residual malignancy in the lung parenchyma. Failure to localize the pulmonary nodule during u-VATS occurred in 5 out of 125 patients, all attributed to incorrect radiotracer placement: in 3 patients, ^99m^Tc-MAA was dislocated within the lung parenchyma; in 2 patients, it spread into the pleural cavity; and in 1 patient, it was inadvertently injected into the thoracic wall. There were no statistically significant differences between the two imaging techniques regarding localization failures.

Pathological examination confirmed radical excision with negative surgical margins in 120 out of 125 cases (96%). The final diagnoses included lung cancer in 60 (50%) patients, metastatic disease in 39 (32.5%), carcinoid tumors in 2 (1.7%), hemangioendothelioma in 1 (0.8%), paraganglioma in 1 (0.8%), and benign lesions in 17 patients (14.2%). Once again, no significant differences were seen between the planar and SPECT/CT imaging in these pathological findings. Neither mortality nor morbidity related to the overall procedure was observed.

## 4. Discussion

Advancements in surgical techniques have facilitated the widespread adoption of minimally invasive approaches, such as VATS, which offers well-documented advantages over open thoracotomy, including reduced invasiveness, faster recovery, and lower perioperative morbidity [7]. Previous studies have demonstrated that ROLL for non-palpable pulmonary lesions during VATS is effective, safe, and cost-efficient, achieving detection rates as high as 98% [23]. These benefits have been observed across different VATS techniques, including multiport, uniport, and robotic-assisted approaches [19,24,25]. Despite high surgical detection rates, planar imaging alone remains suboptimal in accurately assessing radiotracer placement within pulmonary nodules. This limitation stems from the frequent occurrence of radiotracer spillage after ^99m^Tc-MAA injection, which complicates the verification of radiotracer localization within the target nodule or its immediate vicinity. In our case series, planar imaging detected spillage in approximately 46% of cases. These findings highlight the need for advanced imaging modalities to enhance procedural accuracy and optimize patient outcomes. The integration of SPECT/CT has shown promise in refining ROLL by improving the verification of radiotracer placement, even in the presence of spillage, thereby reducing the likelihood of misclassification. Indeed, in our experience, spillage with SPECT/CT was equal to 44%. However, limited data exist on the impact of SPECT/CT imaging in this context. In 2019, Durmo et al. introduced an innovative fluoroscopy system that enabled direct CT-guided radiotracer injection with SPECT/CT, improving procedural efficiency, yet without assessing its role in confirming ^99m^Tc-MAA localization within pulmonary nodules [19]. Similarly, in our previous study, we suggested a potential benefit of SPECT/CT in verifying radiotracer presence, but the small sample size (n = 24 patients) and lack of supporting literature warranted further investigation [20]. In our current study, which includes the largest patient cohort to our knowledge (n = 65 patients), we found a poor concordance (14%) between planar and SPECT/CT imaging in classifying radiotracer placement and spillage. SPECT/CT correctly reclassified radiotracer positioning within the pulmonary nodule in over 40% of cases compared to planar imaging. The subgroup most impacted by SPECT/CT was initially classified as “multisite with focal uptake”, whereas spillage in planar imaging obscured accurate localization. SPECT/CT not only improved the identification of correct radiotracer placement despite spillage but also enhanced the detection of spillage sites, particularly within the bronchial tree, where involvement increased from 4% with planar imaging to 24% with SPECT/CT. A key finding of our study is the potential of SPECT/CT to prevent localization errors during surgical time. In one case, planar imaging suggested a successful procedure based on focal uptake, but SPECT/CT revealed incorrect radiotracer placement in the thoracic wall rather than the pulmonary nodule. This reclassification led to the procedure being deemed unsuccessful, resulting in postponed surgery. Interestingly, pneumothorax was more frequently detected with SPECT/CT than with planar imaging (43% vs. 1.6%; *p <* 0.0001), a discrepancy from the 7–8% reported in the previous literature [24,26]. This difference may be attributed to the additional CT scan performed approximately 10 min after the CT-guided radiotracer injection, a step included only in the SPECT/CT protocol and not in the planar imaging protocol. This additional scan allows more time for pneumothorax detection, suggesting that the complication rates may be higher than traditionally reported. Notably, all cases of pneumothorax were mild and asymptomatic, requiring no intervention.

Although the SPECT/CT group included nodules with a significantly larger size and a higher proportion of subsolid lesions, these differences were not the result of selection bias. The adoption of SPECT/CT imaging was protocol-driven and implemented systematically for all eligible patients from January 2023 onward, regardless of nodule morphology or complexity. Therefore, the observed baseline differences are attributable to random temporal variation rather than intentional allocation criteria.

Despite its advantages, SPECT/CT has some notable limitations. First, it slightly increases procedural time compared to planar imaging (39 min vs. 30 min; *p* < 0.005), which could impact workflow efficiency in high-volume settings. Then, the additional CT scan increases radiation exposure, necessitating careful consideration, particularly for patients requiring repeated diagnostic procedures. A balance must be struck between the diagnostic benefits and potential radiation risks.

The retrospective nature of this study, its short median follow-up, and the lack of a cost-effectiveness analysis represent significant limitations. Considering the marginal impact of SPECT/CT on primary surgical outcomes, its use could be restricted to selective scenarios, such as technically challenging nodules (e.g., deep, subsolid, or close to fissures) or cases where planar images reveal extensive spillage. This approach may offer the best risk–benefit ratio.

## 5. Conclusions

The integration of SPECT/CT imaging into the ROLL procedure for the detection of pulmonary nodules before uVATs shows a significant ability to reclassify radiotracer positioning compared to planar imaging, particularly in cases complicated by radiotracer spillage. However, considering its limited impact on overall surgical success rates and the additional time required, SPECT/CT should be used only in technically challenging cases, such as nodules in complex anatomical locations or those with extensive spillage on planar imaging. Larger sample sizes, multicentric and prospective randomized studies, and formal cost–utility analyses are warranted.

## Figures and Tables

**Table 1:** (**A**) Clinical and demographic characteristics of the patients; (**B**) comparison of baseline characteristics between planar and planar + SPECT/CT groups.

**(A)**			
Patients Characteristics		N = 125	
Median Age (min–max)		67 (33–84)	
Sex (male/female)		55/71	
Nodule Characteristic			
Median Size in mm (range)		12 (5–30)	
Median Volume in cm^3^ (range)		13.5 (6.5–141.3)	
Median Depth in mm (range)		15 (2–65)	
Median ^99m^TC-MAA Activity in MBq (range)		70 (38–210)	
Density, n (%)			
GGO		14 (11.2%)	
psGGO		15 (12%)	
Solid		96 (76.8%)	
Localization, n (%)			
RIL		28 (22.4%)	
LIL		23 (18.4%)	
ML		11 (8.8%)	
RUL		35 (28%)	
LUL		28 (22.4%)	
**(B)**			
**Patients Characteristics**	**Planar Technique Population (N** **=** **60)**	**SPECT Technique Population (N** **=** **65)**	***p*** **-Value**
Median Age (range)	66 (44–81)	68 (33–84)	0.28
Sex (male/female)	28/32	27/38	0.70
Nodule Characteristic			
Median Size in mm	9	12	<0.001
Median Volume in cm^3^	38.2	90.5	<0.001
Nodule Depth, mean in mm	16.1	13.7	0.09
Density, n (%)			
GGO	4 (6.7%)	10 (15.4%)	
psGGO	2 (3.3%)	13 (20%)	
Solid	54 (90%)	42 (64.6%)	

Abbreviations: GGO: ground glass opacity; LIL: left inferior lobe; LUL: left upper lobe; Max: maximum; Min: minimum; ML: medium lobe; psGGO: partial solid ground glass opacity; RIL = right inferior lobe; RUL: right upper lobe.

**Table 2:** Differences between planar and SPECT/CT imaging in 65 patients for whom both planar and SPECT/CT techniques were performed.

	Planar Imaging (n = 60)	SPECT Imaging (n = 65)	Statistical Significance
Timing (mean hours/minutes)			
Nuclear Medicine Service	00:30	00:39	*p* < 0.005
Surgical excision	1:47	2:25	*p* < 0.005
Spillage, n (%)			*p* < 0.0001
Pleural	29 (48%)	11 (17%)	
Subcutaneous	0	1 (1.5%)	
Bronchial tract	1 (1.6%)	16 (24.6%)	
Gastrointestinal tract None	0 30 (50.4%)	1 (1.5%) 36 (55.4%)	
Nuclear Medicine complications, n (%)			
Pneumothorax	1 (1.6%)	28 (43%)	*p* < 0.0001
Hematoma None	0 59 (98.7%)	1 (1.5%) 36 (55.5%)	
Radio-guided intraoperative localization detection rate, n (%) No Yes	1 (1.6%) 59 (98.7%)	4 (6.1%) 61 (93.9%)	NS
Histological Diagnosis, n (%)			
Benign	8 (13%)	11 (17%)	NS
Malignant	52 (87%)	54 (83%)	

## Data Availability

The original contributions presented in this study are included in the article. Further inquiries can be directed to the corresponding author.

## References

[1] Adams S.J., Stone E., Baldwin D.R., Vliegenthart R., Lee P., Fintelmann F.J. (2023). Lung Cancer Screening. Lancet.

[2] de Koning H.J., van der Aalst C.M., de Jong P.A., Scholten E.T., Nackaerts K., Heuvelmans M.A., Lammers J.W.J., Weenink C., Yousaf-Khan U., Horeweg N. (2020). Reduced Lung-Cancer Mortality with Volume CT Screening in a Randomized Trial. N. Engl. J. Med..

[3] Gould M.K., Tang T., Liu I.-L.A., Lee J., Zheng C., Danforth K.N., Kosco A.E., Di Fiore J.L., Suh D.E. (2015). Recent Trends in the Identification of Incidental Pulmonary Nodules. Am. J. Respir. Crit. Care Med..

[4] Xiao J., Li M., Du Q., Han H., Ge Y., Park H. (2021). The Value of Positron Emission Tomography Computed Tomography in Predicting Invasiveness of Ground Glass Nodules: A Protocol for Systematic Review and Meta-Analysis. Medicine.

[5] Mazzone P.J., Lam L. (2022). Evaluating the Patient with a Pulmonary Nodule: A Review. JAMA.

[6] Montagne F., Guisier F., Venissac N., Baste J.-M. (2021). The Role of Surgery in Lung Cancer Treatment: Present Indications and Future Perspectives-State of the Art. Cancers.

[7] Li Y., Dai T. (2023). Meta-Analysis Comparing the Perioperative Efficacy of Single-Port Versus two and Multi-Port Video-Assisted Thoracoscopic Surgical Anatomical Lung Resection for Lung Cancer. Medicine.

[8] Tamura M., Oda M., Fujimori H., Shimizu Y., Matsumoto I., Watanabe G. (2010). New Indication for Preoperative Marking of Small Peripheral Pulmonary Nodules in Thoracoscopic Surgery. Interact. Cardiovasc. Thorac. Surg..

[9] Mattioni G., Palleschi A., Mendogni P., Tosi D. (2022). Approaches and Outcomes of Robotic-assisted Thoracic Surgery (RATS) for Lung Cancer: A Narrative Review. J. Robot. Surg..

[10] Tang L., Zhang Y., Wang Y. (2022). Intraoperative Identification of Pulmonary Nodules During Minimally Invasive Thoracic Surgery: A Narrative Review. Quant. Imaging Med. Surg..

[11] Tsoumakidou G., Saltiel S., Villard N., Duran R., Meuwly J.Y., Denys A. (2021). Image-Guided Marking Techniques in Interventional Radiology: A review of Current Evidence. Diagn. Interv. Imaging.

[12] Lin M.-W., Chen J.-S. (2016). Image-Guided Techniques for Localizing Pulmonary Nodules in Thoracoscopic Surgery. J. Thorac. Dis..

[13] Liu B., Gu C. (2020). Expert consensus Workshop Report: Guidelines for Preoperative Assisted Localization of Small Pulmonary Nodules. J. Cancer Res. Ther..

[14] Imperatori A., Nardecchia E., Cattoni M., Mohamed S., Di Natale D., Righi I., Mendogni P., Diotti C., Rotolo N., Dominioni L. (2021). Perioperative Identifications of Non-Palpable Pulmonary Nodules: A Narrative Review. J. Thorac. Dis..

[15] Chella A., Lucchi M., Ambrogi M.C., Menconi G., Melfi F.M.A., Gonfiotti A., Boni G., Angeletti C.A. (2000). A pilot study of the role of TC-99 radionuclide in localization of pulmonary nodular lesions for thoracoscopic resection. Eur. J. Cardio Thoracic Surg..

[16] Bellomi M., Veronesi G., Trifirò G., Brambilla S., Bonello L., Preda L., Casiraghi M., Borri A., Paganelli G., Spaggiari L. (2010). Computed tomography-guided preoperative radiotracer localization of nonpalpable lung nodules. Ann. Thorac. Surg..

[17] Grogan E.L., Jones D.R., Kozower B.D., Simmons W.D., Daniel T.M. (2008). Identification of Small Lung Nodules: Technique of Radiotracer-Guided Thoracoscopic Biopsy. Ann. Thorac. Surg..

[18] Conte M., De Feo M.S., Frantellizzi V., Tomaciello M., Marampon F., Evangelista L., Filippi L., De Vincentis G. (2023). Radio-Guided Lung Surgery: A Feasible Approach for a Cancer Precision Medicine. Diagnostics.

[19] Durmo R., Lechiara M., Benetti D., Rodella C., Camoni L., Albano D., Bertagna F., Giubbini R. (2019). Radioguided lung lesion localization: Introducing a fluoroscopy system in a SPECT/CT scan. Nucl. Med. Commun..

[20] Aricò D., Macrì P., Bambaci M., Leone G., Romano D., Barbagallo F., Maria S., Picone A., Carnaghi C., Piazza D. (2024). Non-palpable Pulmonary Nodules and Uniportal-VATS: Radio-Guided Localization (ROLL) Experience of a Lung Multidisciplinary Team. Anticancer Res..

[21] Yue X., Cui J., Huang S., Liu W., Qi J., He K., Li T. (2025). An interpretable radiomics-based machine learning model for predicting reverse left ventricular remodeling in STEMI patients using late gadolinium enhancement of myocardial scar. Eur. Radiol..

[22] Zhan Y., Song F., Zhang W., Gong T., Zhao S., Lv F. (2024). Prediction of benign and malignant pulmonary nodules using preoperative CT features: Using PNI-GARS as a predictor. Front. Immunol..

[23] Ricciardi S., Davini F., Manca G., De Liperi A., Romano G., Zirafa C.C., Melfi F. (2020). Radioguided Surgery, a Cost-Effective Strategy for Treating Solitary Pulmonary Nodules: 20-Year Experience of a Single Center. Clin. Lung Cancer.

[24] Davini F., Ricciardi S., Zirafa C.C., Cavaliere I., Romano G., Melfi F. (2018). Treatment of Pulmonary Nodule: From VATS to RATS. J. Vis. Surg..

[25] Manca G., Davini F., Tardelli E., De Liperi A., Falaschi F., Melfi F., Colletti P.M., Rubello D., Volterrani D., Boni G. (2018). Clinical Impact of Radioguided Localization in the Treatment of Solitary Pulmonary Nodule: A 20-Year Retrospective Analysis. Clin. Nucl. Med..

[26] Carvajal C., González F., Beltrán R., Buitrago R., Reyes A.d.L., Llamas A., Beltrán J., Carreño J. (2021). Lung nodule radio-guided localization and uniportal video-assisted thoracoscopic surgery resection. Updat. Surg..

